# Walrus population-specific marine reservoir offsets (ΔR) for calibration of radiocarbon dates: Implications for Arctic chronologies and medieval trade

**DOI:** 10.1177/09596836261468295

**Published:** 2026-08-15

**Authors:** Bente Philippsen, Katrien Dierickx, Mohsen Falahati-Anbaran, Erin H. Kunisch, Thomas C. A. Royle, Richard Sabin, Eirik Sollid, James H. Barrett

**Affiliations:** 1 NTNU University Museum, Trondheim, Norway; 2 Natural History Museum, London, UK

**Keywords:** radiocarbon dating, marine reservoir correction, walrus, natural history collections, trade, Arctic, ivory, Medieval

## Abstract

Walruses have long played a vital role in Arctic subsistence and commercial economies, yet accurate radiocarbon dating of walrus-derived archaeological materials is complicated by regional variation in marine reservoir effects. This study presents new ΔR values derived from 31 new and 15 legacy radiocarbon dates on known-age walrus specimens spanning multiple populations of *Odobenus rosmarus rosmarus* and *O. r. divergens*. The results reveal substantial inter-population variability, with ΔR values ranging from −140 (Franz Josef Land) to +295 (Pacific), underscoring the importance of population-specific calibration. A pooled ΔR of +17±12 was calculated for western Greenland and the Canadian Arctic—regions central to the medieval Norse ivory trade—and applied to 12 walrus rostra and ivory artefacts excavated in Trondheim, Norway. The resulting calibrated dates are from the 11th to early 14th centuries CE, confirming that seemingly anomalous post-15th-century finds are residual rather than evidence of continued trade. An alternative ΔR estimate of −110 ± 35, derived solely from archaeological context dates, suggests potential time lags between harvest and deposition. These findings demonstrate the value of known-age walrus ΔR data for refining chronologies of Arctic exploitation and long-distance trade, while highlighting the need for provenance studies in archaeological dating and both the pertinence and limitations of mollusc-based reservoir corrections.

## Introduction

Walruses have been an important resource for northern communities for centuries. They provided meat, blubber, hide and/or ivory (Frei et al., 2015; Keighley et al., 2021; Mason and Rasic, 2019). Today, they remain a valued food item and focus of small-scale subsistence hunts among Chukchi, Inuit, and Yupik communities in Greenland, Arctic Canada, Alaska and Russia (Born et al., 2017; Desjardins and Gotfredsen, 2021; Metcalf et al., 2025; Zdor et al., 2010). Historically, walruses were commercially exploited on a large scale by European hunters, whalers and settlers. This led to severe population declines and in some instances extirpations (e.g. Barrett et al., 2020; Gjertz, 2021; Keighley et al., 2019; Kovacs et al., 2014; McCaffrey, 2020; McLeod et al., 2014).

Research on the cultural role of walrus hunting, human and environmental impacts on walrus populations and their ecosystem often relies on absolute chronologies derived from radiocarbon dating. These dates are calibrated using a calibration curve that accounts for the marine reservoir effect (e.g. Dury et al., 2022; Dyke et al., 2019). However, the standard marine calibration curve, Marine20, represents a global modelled ocean and corrects only for the average reservoir age, R. It is not intended for calibration in polar regions (Heaton et al., 2020).

Reservoir ages vary locally due to factors such as upwelling of older bottom water, inflow of carbonate-rich river water and seasonal ice cover, which limits CO_2_ exchange with the atmosphere. This variability is especially pronounced in the Arctic Ocean, where a stable surface layer and permanent ice cover further restrict exchange with atmospheric CO_2_ (Heaton et al., 2020; Mangerud and Gulliksen, 1975). Conversely, areas with shallow water and better atmospheric exchange may have lower reservoir ages. The local deviation from the global modelled reservoir age is denoted ΔR and is essential for accurate calibration of samples of unknown age.

To estimate ΔR, known-age samples from specific localities are dated so that their local offset can be calculated. Samples collected before the 1950s are preferred to avoid effects from the ^14^C excess produced by atomic bomb tests. In this paper, we discuss and calculate ΔR values respective to the Marine20 curve, despite its limited suitability for polar regions, to ensure compatibility with recent publications and databases, such as the 14CHRONO Marine20 Reservoir database (calib.org/marine, Reimer and Reimer, 2001). When we refer to ΔR values respective to other marine calibration curves, we will specify this, for example, as ΔR_Marine13_.

Relatively extensive ΔR datasets are available from mollusc shells, as these are easy to collect, curate and sample after decades of storage (e.g. Pearce et al., 2023; Pieńkowski et al., 2023; Reimer and Reimer, 2001). However, mollusc shells may not be representative of walrus diet, even when molluscs dominate the diet. The flesh of the molluscs can have ΔR values that differ from those of their shells (Erlenkeuser et al., 1975: 288–290; Fernandes et al., 2012). Walrus dentine and bone collagen are formed from dietary protein rather than shell carbonate (Dyke et al., 2019; Jim et al., 2004). For example, the δ^13^C value of mollusc shell was shown to change throughout ontogeny in one species, indicating shifts in carbon source for shell formation (Lorrain et al., 2004). The carbon in the flesh derives from the mollusc’s diet, while the majority of the carbon in the shell carbonate derives from water dissolved inorganic carbon or DIC (McConnaughey and Gillikin, 2008). In many cases, the age difference should not be significant, as the diet of for example, filter feeders may be dominated by phytoplankton, whose DIC source is the same as that for the mollusc shell (Gillikin et al., 2006). In this paper, we radiocarbon date bones of walruses with known years of death to avoid such possible ambiguities. These walruses are from different populations or regional groupings previously identified on the basis of genetic and other evidence (e.g. Dierickx et al., 2026b; Keighley et al., 2022; Ruiz-Puerta et al., 2023), to calculate their ΔR. The focus is on populations of the Atlantic subspecies, *Odobenus rosmarus rosmarus*, for which 43 known-age specimens pre-dating 1950 were available in natural history museums for radiocarbon dating (comprising 29 new and 15 previously studied samples, with one specimen sampled twice). The resulting ΔR values are divided by population where practicable. A pooled value for Greenland (excluding East Greenland) and the Canadian Arctic is also calculated for use with walrus artefacts traded to Europe for which a precise location of harvest cannot be inferred. For comparison with future work, a more tentative ΔR is also provided for the Pacific subspecies, *Odobenus rosmarus divergens*, based on two known-age specimens.

As a case study, we employ the ΔR results to interpret the chronology of medieval artefacts of walrus rostrum (skull) bone and tusk ivory recovered from archaeological contexts in Trondheim, Norway, and its hinterland. Trondheim was one of several major hubs for the trade and carving of walrus tusks, which were traded as pairs attached to the rostrum bone (Barrett, 2021; Barrett and Grav, 2025). Ancient DNA (aDNA) and stable isotope evidence shows that the material originated in western Greenland and/or Arctic Canada, indicating that it was acquired from or via the Norse colony of Greenland (Barrett et al., 2020; Ruiz-Puerta et al., 2024). Two questions are asked: (a) whether the pooled ΔR for Greenland and the Canadian Arctic based on known age material from natural history collections matches a ΔR estimated based on stratified archaeological finds from Trondheim and (b) whether walrus artefacts from the town that seem to post-date the 15th-century Norse abandonment of Greenland based on archaeological evidence are residual finds from earlier centuries, or if existing interpretations of the chronology of the Greenlandic trade in walrus ivory should instead be reassessed?

## Materials and methods

Samples of bone from 31 known-age walrus specimens pre-dating 1950 were collected from the American Museum of Natural History (New York, NY, USA; *n* = 10), the Canadian Museum of Nature (Ottawa, ON, CA, and Gatineau, QC, CA; *n* = 4), the Natural History Museum (London, UK; *n* = 7) and the Zoological Museum, Natural History Museum of Denmark (Copenhagen, DK; *n* = 10). Locations and kill dates were available from the catalogue records of the relevant museums, and were sometimes also preserved on original labelling (Figures 1a and 2). Placenames indicating catch locations represented past usage, but modern locations could be inferred with confidence (Table 1). The finds derive from Arctic exploration between 1852/53 and 1929, including expeditions by (e.g.) Knud Rasmussen/Peter Freuchen (fifth Thule expedition to Nunavut), F.G. Jackson (Franz Josef Land), Alwin Pedersen (Scoresby Sund/Kangertittivaq) and Robert Peary (north-west Greenland).

**Figure 1.**
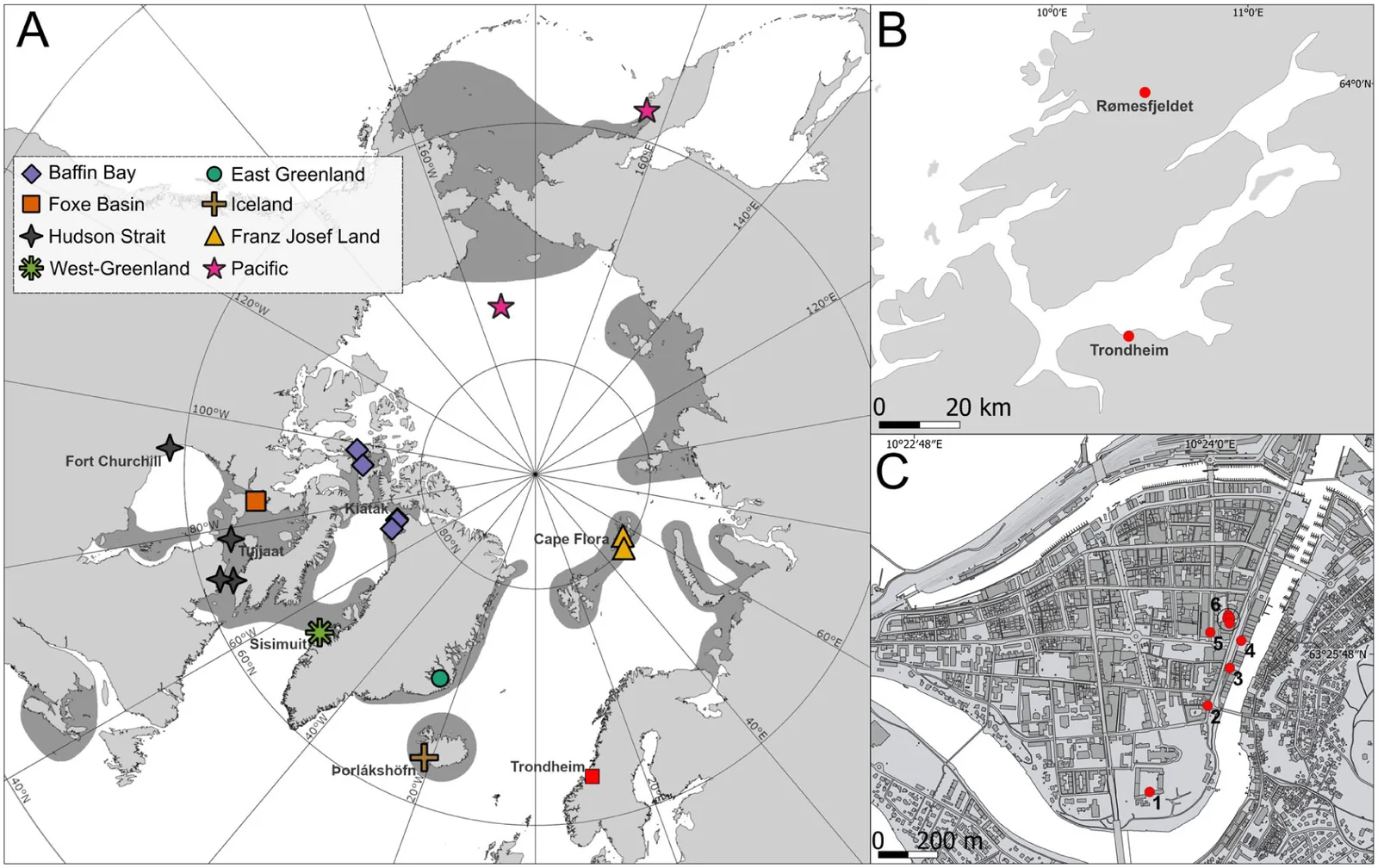
Geographical locations of: (a) newly radiocarbon dated walrus specimens from natural history collections with known date of death, plotted in relation to the modern circumpolar distribution of the species (dark grey); (b) Trondheim and Rømesfjeldet (modern Rømmesfjellet), the latter being the find spot of a walrus tusk radiocarbon dated in this study; (c) excavations within Trondheim yielding finds of walrus rostra and/or walrus ivory that are radiocarbon dated in this study (1 Erkebispegården, 2 Kjøpmannsgata, 3 Kjøpmannsgata Waterfront, 4 Kjøpmannsgata 25–27, 5 Søndre gate, 6 Folkebibliotekstomten). Modern walrus distribution based on Andersen et al. (2014), McLeod et al. (2014), Keighley et al. (2019), Beatty et al. (2020), Keighley et al. (2022) and Mills et al. (2024). Basemaps derived from Natural Earth (www.naturalearthdata.com/) and the *Felles kartdatabase* of the Norwegian Mapping Authority (Statens kartverk).

**Figure 2.**
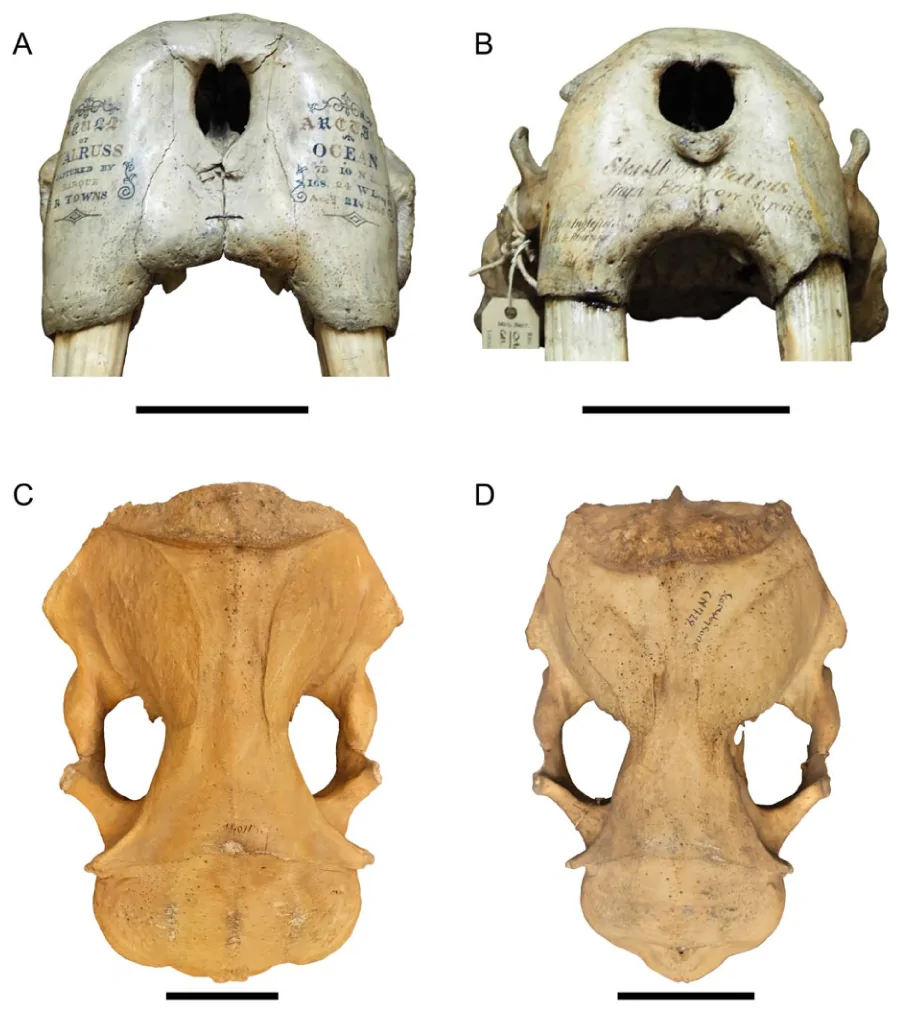
Examples of walrus specimens from natural history collections having known dates of death: TRa numbers 22951 ((a) NHMUK GERM 331.j) and 22943 ((b) NHMUK ZD.1855.11.26.38) from the collections of the Natural History Museum, London, 25065 ((c) AMNH MO-11049) from the collections of the American Museum of Natural History and 25588 ((d) NHMD-M11-CN729) from the collections of the Natural History Museum of Denmark (photographs by James Barrett (a and b) and Katrien Dierickx (c and d). Scale bars indicate 10 cm.

**Table 1.** New radiocarbon dates on walrus specimens with historical dates of death.

Lab number (TRa-)	Population	Origin as historically catalogued	Origin	Date of death CE	^14^C Age (yr BP)	^14^C error (1σ)
22945	Baffin Bay	Wellington Channel, Nunavut	Wellington Channel	1852–1853	649	12
22943	Baffin Bay	Barrow Strait, Nunavut	Barrow Strait	1853–1854	725	15
22944	Baffin Bay	Barrow Strait, Nunavut	Barrow Strait	1854	737	13
25066	Baffin Bay	Murchison Sound, Greenland	Murchison Sund	1895	582	13
25068	Baffin Bay	Manson Island, Greenland	Qeqertaarsuit (Manson Øer)	1896	603	14
25065	Baffin Bay	Manson Island, Greenland	Qeqertaarsuit (Manson Øer)	1896	599	15
25069	Baffin Bay	Manson Island, Greenland	Qeqertaarsuit (Manson Øer)	1896	582	13
25064	Baffin Bay	Murchison Sound, Greenland	Murchison Sund	1896	535	13
25063	Baffin Bay	Murchison Sound, Greenland	Murchison Sund	1896	569	21
25062	Baffin Bay	Northumberland Island, Greenland	Kiatak	1926	565	18
25061	Baffin Bay	Northumberland Island, Greenland	Kiatak	1926	563	13
25060	Baffin Bay	Northumberland Island, Greenland	Kiatak	1926	553	16
25067	Western Greenland	Holsteinsborg, Greenland	Sisimiut	1895	611	20
25591	Western Greenland	Holsteinsborg, Greenland	Sisimiut	1898	604	13
25595	Foxe Basin	Frozen Strait, Nunavut	Frozen Strait	1922	646	18
25596	Foxe Basin	Frozen Strait, Nunavut	Frozen Strait	1922	687	16
24149	Hudson Strait/ Bay	Nottingham Island, Nunavut	Tujjaat	1884–1885	671	20
24150	Hudson Strait/ Bay	Prince of Wales Sound, Hudson Strait, Nunavut	Hudson Strait	1884–1885	639	13
24148	Hudson Strait/ Bay	Ashe Inlet, Hudson Strait, Nunavut	Ashe Inlet	1886	609	12
24151	Hudson Strait/ Bay	Fort Churchill, Manitoba	Churchill	1910	615	12
25589	Eastern Greenland	North-east Greenland	Tunu	1906–1909	631	21
25593	Eastern Greenland	Scoresby Sund, Greenland	Kangertittivaq	1924	569	13
25587	Eastern Greenland	Scoresby Sund, Greenland	Kangertittivaq	1927	605	23
25588	Eastern Greenland	Scoresby Sund, Greenland	Kangertittivaq	1927–1929	558	16
25585	Eastern Greenland	Scoresby Sund, Greenland	Kangertittivaq	1929	561	16
25586	Eastern Greenland	Scoresby Sund, Greenland	Kangertittivaq	1929	567	15
25590	Iceland	Þorlákshöfn, Iceland	Þorlákshöfn	1900	576	14
22952	Franz Joseph Land	Cape Flora, Franz Josef Land	Cape Flora	1894–1897	472	15
22947	Franz Joseph Land	Franz Josef Land	Franz Josef Land	1894–1897	494	15
22951	Pacific	Arctic Ocean (75°10ʹN Lat; 168°24ʹW Long)	Arctic Ocean	1865	843	13
22950	Pacific	Kamtchatka	Kamchatka	1896–1897	1026	16

Full specimen details and museum catalogue numbers are available in Supplemental Material Table S1. European place-names are retained if needed to avoid ambiguity.

For the Trondheim case study, six samples of walrus skull (rostrum) bone and six samples of walrus ivory (one crucifix corpus, one gaming piece, three offcuts of raw material and one complete tusk) were obtained from archaeological collections curated at the NTNU University Museum (Trondheim, NO; Table 2, Figure 3). The rostra represent the original ‘packaging’ in which pairs of tusks were traded from Greenland in the Middle Ages (Barrett et al., 2020). Eleven of the Trondheim area finds were from within the medieval town itself, with the last sample being from one of two tusks (from the same animal) bearing owner’s markings that were found at Rømesfjeldet (Rømmen, Åfjorden) north of the town (Figure 1b and c).

**Table 2.** Radiocarbon dated walrus specimens from excavations in Trondheim and its hinterland.

Lab number (TRa-)	Museum number	Archaeological site	Object	Archaeological context (CE)	Dating as used in the model (C_Date ± 1σ)	Context	^14^C Age (yr BP)	^14^C error (1σ)
25180	N10102	Søndre gate	Rostrum fragment	1111 or shortly before	1111 ± 1	In situ	1399	16
25175	N37603	Folkebibliotekstomten	Rostrum	post-1600		Residual?	1389	21
25176	N32091	Folkebibliotekstomten	Rostrum fragment	1225–1275	1250 ± 13	In situ	1388	14
25886	N33186	Folkebibliotekstomten	Tusk (gaming piece)	1100–1150	1125 ± 13	In situ	1364	34
25178	N29494	Folkebibliotekstomten	Rostrum	1150–1175	1163 ± 6	In situ	1363	20
25885	N41911	Kjøpmannsgata 25–27	Tusk offcut	undated		Unstratified	1351	12
25884	N30109a	Folkebibliotekstomten	Tusk offcut	1175–1225	1200 ± 13	In situ	1321	12
25888	T2383:2	Rømesfjeldet, Åfjorden	Tusk	undated		Unstratified	1313	14
25179	N203145	Kjøpmannsgata Waterfront	Rostrum fragment	12th century	1150 ± 25	In situ	1312	13
25887	T4869	Kjøpmannsgata	Tusk (crucifix)	undated		Unstratified	1255	11
25177	N167587	Erkebispegården	Rostrum	1500–1532		Residual?	1229	13
25883	N19934	Folkebibliotekstomten	Tusk offcut	1225–1275	1250 ± 13	In situ	1226	13

Full specimen details are available in Supplemental Material Table S2. The dates are here calibrated (95.4% range) with the ΔR of +17 ± 12 calculated for historical specimens from Greenland (excluding Eastern Greenland) and the Canadian Arctic.

**Figure 3.**
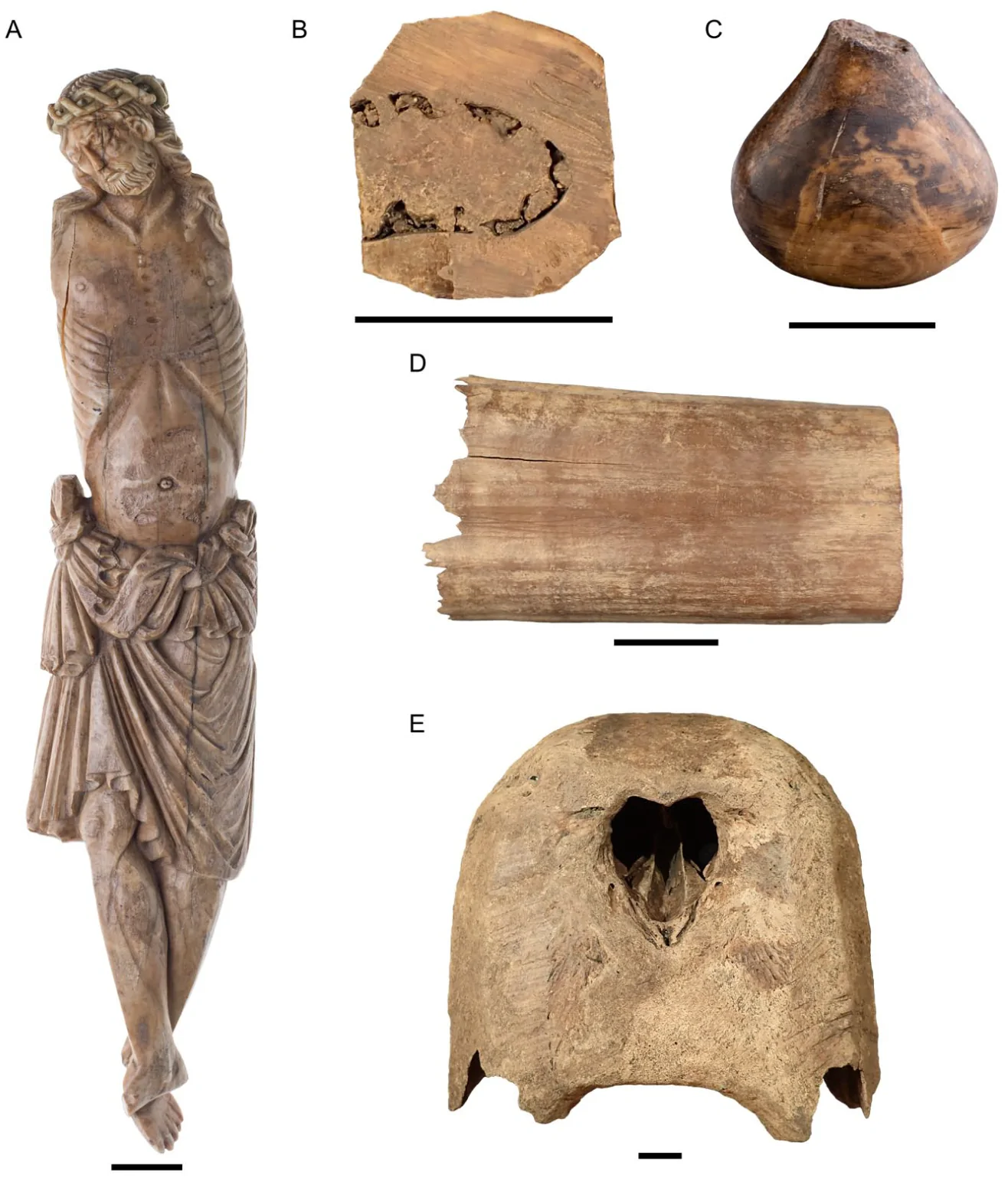
A selection of the medieval walrus ivory artefacts and rostrum bone from Trondheim radiocarbon dated in this study: museum numbers T4869 ((a) crucifix), N30109a ((b) tusk offcut), N33186 ((c) gaming piece), N41911 ((d) tusk offcut), N37603 ((e) rostrum), (photographs by Åge Hojem (a and c) and J.H. Barrett (b, d and e), NTNU University Museum). Scale bars indicate 2 cm.

The newly studied known-age natural history specimens were sampled using a Dremel rotary tool, either by cutting a small chunk from a cranium using a diamond rotary blade (*n* = 28) or drilling powder from a mandibular tooth socket using a steel bit and/or a diamond bur (*n* = 3). Most (*n* = 19) of the cranial samples were cut from tympanic bullae, with the remainder (*n* = 9) taken from other locations (mastoid, parietal, temporal, tusk socket or other maxillary bone) depending on the completeness of the specimens and the guidance of museum curators. Original samples were typically between 0.5 and 1 g, as agreed with the relevant museum and/or heritage authority. The archaeological samples from the NTNU University Museum were also sampled with a Dremel rotary tool using a diamond rotary blade (to cut a small chunk) or diamond burrs and steel drill bits (to extract powder). For these artefacts, curatorial considerations sometimes necessitated smaller samples of c. 0.3–0.4 g. Both the natural history and archaeological samples were used for multiple analyses (e.g. isotope analysis and ancient DNA (aDNA)) of which the radiocarbon dating reported here was one. Potentially contaminated (e.g. dusty) external surfaces were abraded or cut away prior to on-site sampling or in-lab subsampling.

Collagen was extracted from the bone or ivory samples with a modified Longin procedure (Longin, 1971) as described in Seiler et al. (2019), with additional modifications to maximise yield and minimise potential contamination. Samples were defatted with alternate washes of a 2:1 (v/v) chloroform:methanol and 2:1 (v/v) methanol:chloroform solution in an ultrasonic bath for a maximum of 15 min each, until the solution remained clear. Another modification to the protocol described in Seiler et al. (2019) was the adjustment of temperature and concentration. Demineralisation was done in 0.2 to 0.5M HCl at 4°C until complete (24 h to c. 4 weeks, depending on the sample particle size and preservation). Humic substances were removed with 0.1 M NaOH at 4°C, left overnight and repeated until the solution remained clear. Between/after reagents, samples were washed three times with ultrapure water until neutral. The samples were gelatinised at 70°C and pH = 3 for about 48 h (or until complete), and freeze-dried. Combustion, graphitisation and measurement was performed at the National Laboratory for Age Determination (Trondheim, NO) as described in Seiler et al. (2019) and Nadeau et al. (2015).

We used OxCal v.4.4.4 (Bronk Ramsey, 2009; Bronk Ramsey and Lee, 2013) to calculate ΔR values for the known-age specimens from natural history collections (Table 3, Figure 4). Two alternative approaches were used. The first method used only date of death information, because it is known with considerable certainty. The second method included both date of death and inferred date of birth. Date of birth was estimated using the osteological age categories of Dierickx et al. (2026a): neonatal (birth to 3/4 years), juvenile (c.4 to c.10 years) and adult (c.10 to c. 40 years); the midpoint of each age category was subtracted from date of death and attributed an error term such that two standard deviations would encompass the full age category. For adults, for example, 25 years was subtracted from date of death and the estimated date of birth then attributed an error of 7.5 (rounded to 8) years. Estimated date of birth was used to constrain the *terminus post quem* (TPQ) of the models because the bone collagen turnover rate for walruses is not well established.

**Table 3.** ΔR values per population (95.4% ranges) based on differing OxCal models and the exclusion or inclusion of legacy radiocarbon dates; n = number of dated specimens.

Population	OxCal constraints	ΔR range from	to	ΔR mean	ΔR 1 σ	Dates included
BAF (*n* = 12)	DoD	−98	+2	−47	24	This study
BAF (*n* = 12)	eDoB & DoD	−66	+14	−26	19	This study
BAF (*n* = 13)	eDoB & DoD	−51	+27	−12	19	This study; Dyke et al. (2019)
WGL (*n* = 2)	DoD	−199	+81	−54	72	This study
WGL (*n* = 2)	eDoB & DoD	−113	+78	−17	47	This study
FOX (*n* = 2)	DoD	−215	+169	+13	95	This study
FOX (*n* = 2)	eDoB & DoD	−31	+156	+62	46	This study
FOX (*n* = 5)	eDoB & DoD	−10	+109	+49	29	This study; Dyke et al. (2019)
HUD (*n* = 4)	DoD	−110	+61	−23	43	This study
HUD (*n* = 4)	eDoB & DoD	−63	+70	+4	33	This study
HUD (*n* = 14)	eDoB & DoD	0	+71	+35	18	This study; Dyke et al. (2019)
Pooled ΔR for Greenland and Canadian Arctic (BAF, WGL, FOX, HUD; *n* = 20)	DoD	−72	+5	−33	19	This study
Pooled ΔR for Greenland and Canadian Arctic (BAF, WGL, FOX, HUD; *n* = 20)	eDoB & DoD	−40	+20	−10	15	This study
Pooled ΔR for Greenland and Canadian Arctic (BAF, WGL, FOX, HUD; *n* = 34)	eDoB & DoD	−9	+42	+17	12	This study; Dyke et al. (2019)
EGL (*n* = 6)	DoD	−119	+20	−48	33	This study
EGL (*n* = 6)	eDoB & DoD	−82	+29	−26	27	This study
ICE (*n* = 1)	DoD	−408	+85	−115	121	This study
ICE (*n* = 1)	eDoB & DoD	−182	+84	−49	66	This study
FJL (*n* = 2)	DoD	−391	−42	−189	82	This study
FJL (*n* = 2)	eDoB & DoD	−235	−45	−140	47	This study
PAC (*n* = 2)	DoD	−103	+387	+179	129	This study
PAC (*n* = 2)	eDoB & DoD	+200	+391	+295	47	This study

BAF: Baffin Bay; WGL: West Greenland; FOX: Foxe Basin; HUD: Hudson Strait and Hudson Bay; EGL: East Greenland; ICE: Iceland; FJL: Franz Josef Land; PAC: Pacific (see Figure 1); DoD: date of death; eDoB: estimated date of birth.

**Figure 4.**
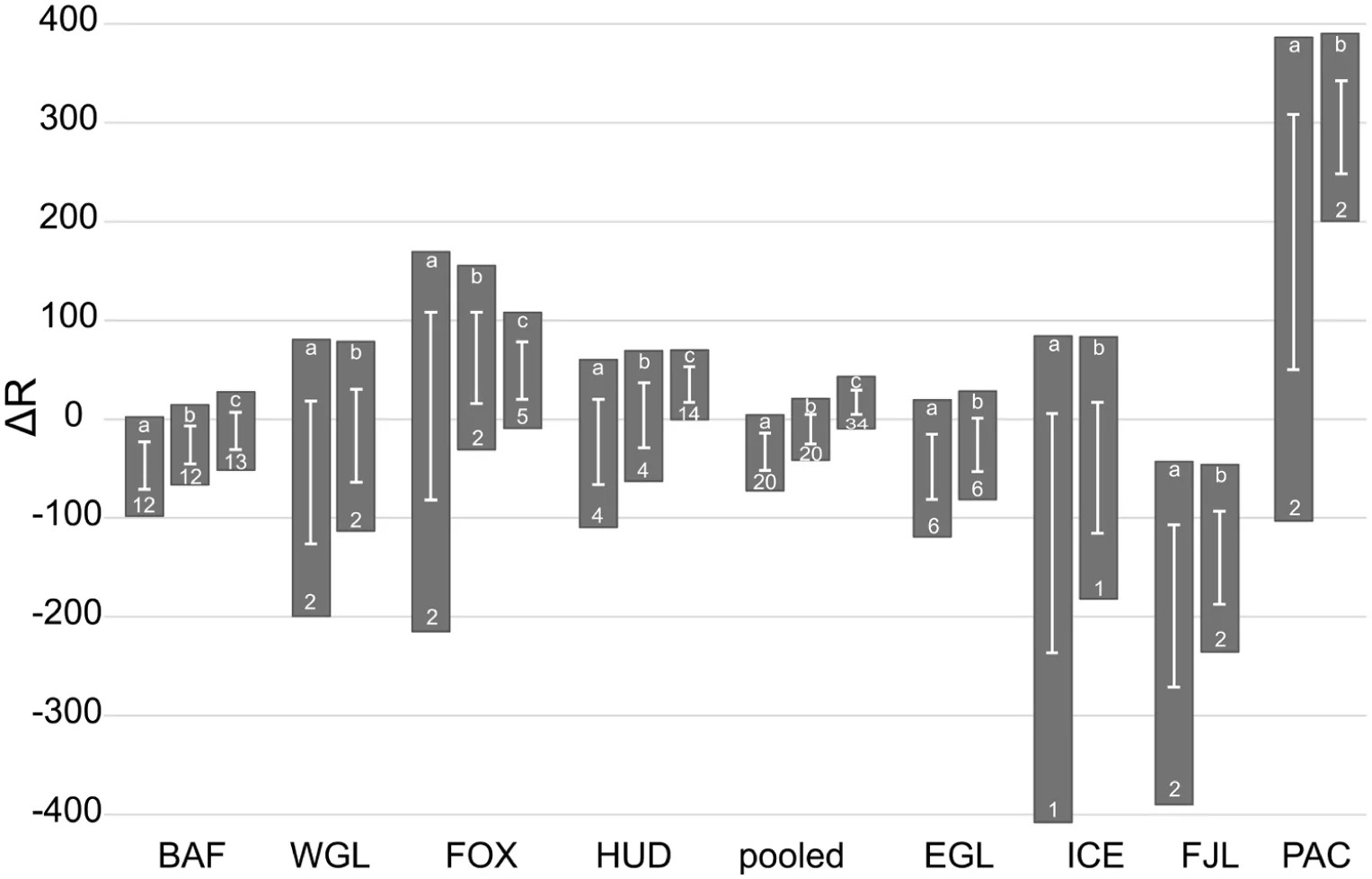
ΔR values per population (95.4% ranges) based on differing OxCal models and the exclusion or inclusion of legacy radiocarbon dates; data from Table 3. BAF: Baffin Bay; WGL: West Greenland; FOX: Foxe Basin; HUD: Hudson Strait and Hudson Bay; EGL: East Greenland; ICE: Iceland; FJL: Franz Josef Land; PAC: Pacific (see Figure 1). The error bar denotes the mean ΔR plus/minus one standard deviation; the box marks the ΔR range (from Table 3). The letters in the upper part of the boxes mark the calculation: (a) only using date of death, (b) additionally using estimated date of birth, (c) using date of death, estimated date of birth and data from Dyke et al. (2019). The numbers in the lower parts of the boxes are the numbers of samples included in these averages.

The radiocarbon dates of these samples were placed in a simple sequence model, where the radiocarbon age (R_Date) was followed by the date of death (C_Date) (method 1) or bracketed by the estimated date of birth (C_Date) and the date of death (C_Date) (method 2). The uncertainty for date of death was set to ±1 year if known to a single year, or increased as appropriate in instances when catch date was only known within several years. In the latter cases, additional years were also added to the error of the estimated date of birth. We calculated ΔRs per population. Furthermore, we calculated a pooled ΔR value for the populations that were potentially relevant for the medieval trade of walrus tusks, that is, Greenland (excluding East Greenland) and the Canadian Arctic. There may be differences in diet and therefore in ΔR between individuals related to the size, age or sex of individuals and specific feeding locations, but the aim of this study is to provide reliable ΔR estimates for specimens for which this information will typically not be available. Therefore, potential differences within populations are not a focus of this paper. As a final step, we used method 2 as described above to calculate ΔR values per population (and a pooled ΔR value for Greenland (excluding East Greenland) and the Canadian Arctic) when also including 15 radiocarbon dates on known-age walrus specimens pre-dating 1950 published by Dyke et al. (2019). One of these previously studied specimens (UCIAMS-168850) was re-examined within the present project (TRa-24150), but the 14 others were not examined in-hand for ontological age assessment. For the purposes of TPQ (C_Date) modelling, the date of birth of the Dyke et al. (2019) specimens was thus estimated as the date of death minus 20 years with a standard deviation of 10 years.

The twelve archaeological samples from Trondheim and its hinterland were calibrated three times. Firstly, we used the pooled ΔR calculated for the historical samples from Greenland and the Canadian Arctic, +17±12, and applied that to all samples. All OxCal code is provided in the Supplemental Material. Secondly, we used the same ΔR but also included archaeological context information as described below.

For the third calibration of the archaeological samples, OxCal was used to estimate an appropriate ΔR in a similar way as for the historical specimens; as several samples originate from relatively well-dated contexts, we could use the date of the context in the same way as the year of death for historical samples (see e.g. Macario et al., 2023; van Den Hurk et al., 2025). For example, if a sample’s context was dated to 1225–1275 CE, we assume a symmetrical distribution of the true calendar age around 1250 CE with ±25 years at 2σ. The C_Date for the model (with 1σ uncertainty) is thus 1250 ± 12.5, rounded to 1250 ± 13. In interpreting the results of this approach, it is necessary to account for the possibility that the year of incorporation into the archaeological record may be later than the year of death of the animal.

Table 4, Figures 5 and 6 display the results of these different approaches. All OxCal code can be found in online Supplemental Material 2, section 2.10.1 and 2.10.2.

**Table 4.** Calibrated date ranges CE (95.4%) for medieval walrus artefacts from Trondheim and its hinterland, calibrated in OxCal v4.4.4 (Bronk Ramsey, 2009) using the Marine20 curve (Heaton et al., 2020) with three different approaches as described in the text.

		Date cal. CE (95.4%; ΔR of 17 ± 12).	Date cal. CE (95.4%; ΔR of 17 ± 12, with archaeological context information)	Date cal. CE (95.4%; ΔR of −110 ± 35, derived from archaeological dates)
Lab number (TRa-)	Archaeological dating cal. CE (95.4%)	OxCal code: suppl.mat. 2, section 2.10.1	OxCal code: suppl.mat. 2, section 2.10.2	OxCal code: suppl.mat. 2, section 2.10.2
25180	1111 or shortly before	1049–1291	1051–1115	963–1115
25176	1225–1275	1056–1300	1128–1276	976–1260
25175	Post-1600 (interpreted as residual)	1055–1302	1045–1291	911–1221
25178	1150–1175	1067–1325	1073–1175	1000–1174
25886	1100–1150	1058–1332	1048–1149	984–1149
25885	undated	1079–1335	1071–1318	969–1259
25884	1175–1225	1134–1386	1114–1228	1038–1219
25888	undated	1144–1395	1118–1378	1012–1282
25179	12th century	1149–1395	1070–1205	1031–1190
25887	undated	1209–1426	1189–1414	1050–1320
25177	1500–1532 (interpreted as residual)	1230–1440	1220–1433	1063–1347
25883	1225–1275	1235–1440	1188–1275	1094–1267

The OxCal code is provided in Supplemental Material File 2 (section 2.10).

**Figure 5.**
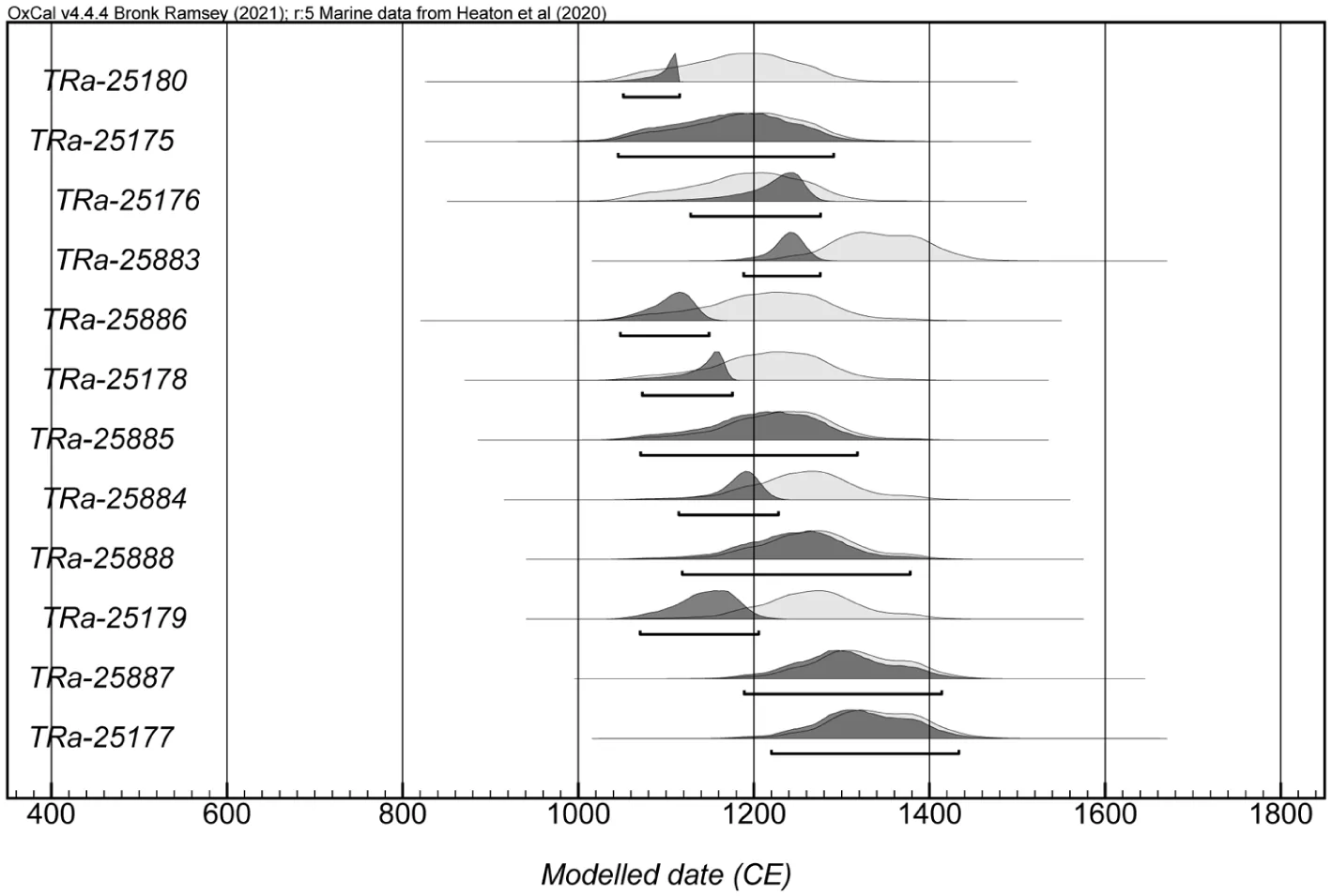
Calibrated ages of archaeological walrus samples from excavations in Trondheim and its hinterland, calibrated in OxCal v4.4.4 (Bronk Ramsey, 2009) using the Marine20 curve (Heaton et al., 2020), the ΔR of 17 ± 12 calculated for historical specimens from Greenland (excluding Eastern Greenland) and the Canadian Arctic and prior information from the dates of the relevant archaeological contexts. See Table 2 for details and Table 4 for calibrated age ranges.

**Figure 6.**
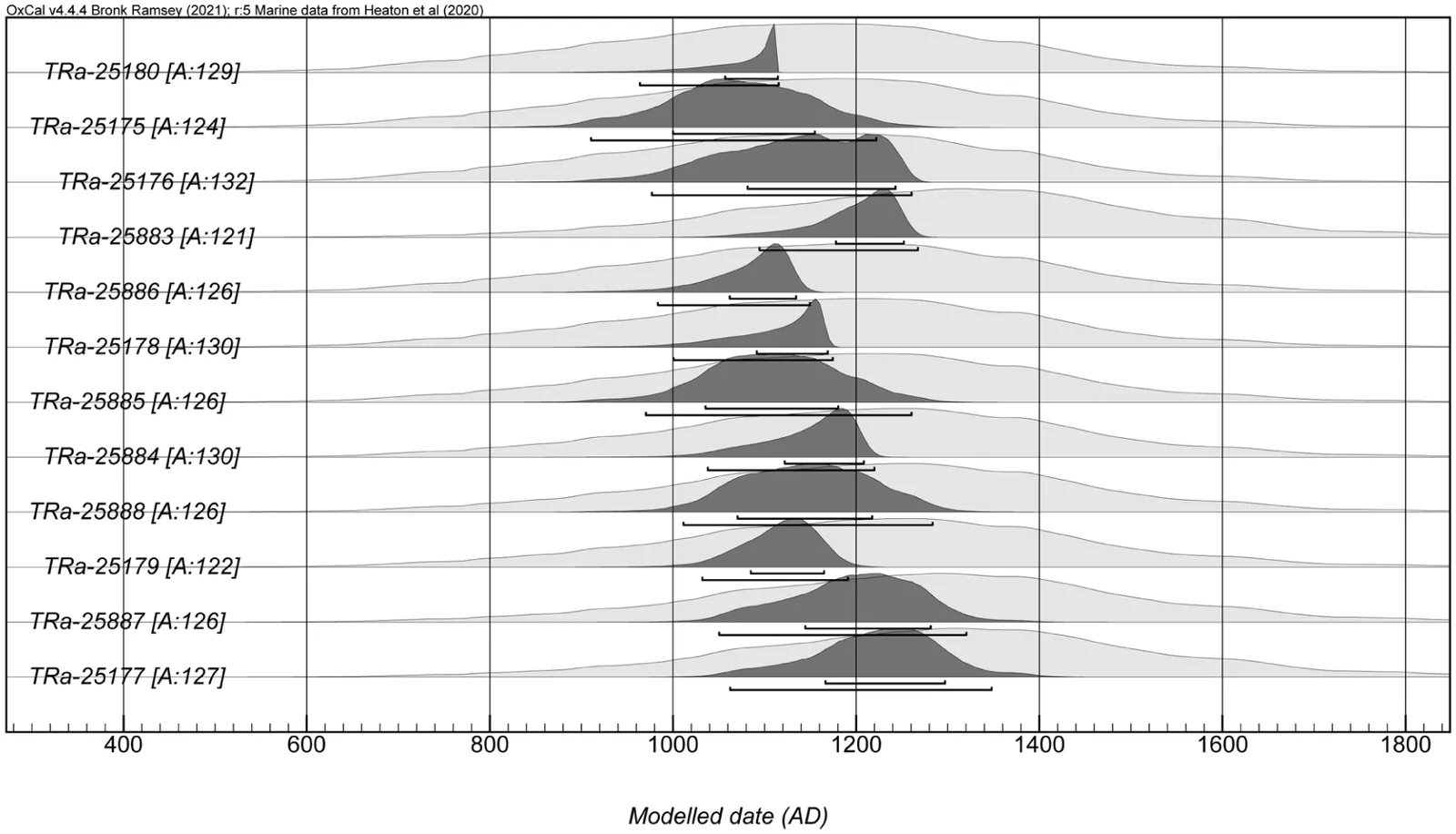
Alternative calibrated ages of archaeological walrus samples from excavations in Trondheim and its hinterland. For this figure, we did not specify a ΔR based on known-age natural history specimens, but let OxCal estimate the most appropriate ΔR based on the archaeological context dating, with a prior of U(−400,400). This resulted in ΔR between −184 and −40 (95.4%), with a median value of −107, mean −110 and standard deviation of 35. See also the last column of Table 4.

## Results and discussion

### ΔR values from known-age Walrus specimens

Based on the known-date natural history specimens, the calculated ΔR values per walrus population and pooled for Greenland (excluding East Greenland) and the Canadian Arctic, are summarised in Table 3. The ΔR estimates calculated using the different methods agree within uncertainties (Table 3, Figure 4). Each estimate is valid within its own assumptions and limitations. Of the three approaches, the ΔR calculations including both estimated dates of birth and legacy dates are employed for the discussion that follows. Although these results entail more assumptions (regarding the age of the individual walruses) than the calculations using only date of death, they provide improved accuracy and precision.

There are considerable differences between the ΔR values for some populations. The mean ΔR values range from −140 for the samples from Franz Josef Land to +295 for the samples from the Pacific. The standard deviations also vary between populations; populations with a larger number of dated specimens have more restricted ΔR values.

The pooled ΔR for Greenland (excluding East Greenland) and the Canadian Arctic is +17 ± 12. This value is heavily influenced by the samples from Baffin Bay (*n* = 13) and Hudson Strait/Hudson Bay (*n* = 14). The Baffin Bay samples alone have a ΔR of −12 ± 19. This is only slightly lower than the value recorded in the 14CHRONO Marine20 Reservoir database at http://calib.org/marine/ (Reimer and Reimer, 2001), drawing mainly on Pearce et al. (2023), where 17 shell samples from Baffin Bay have an average ΔR of +31 ± 97. The 14 samples from the Hudson Strait and Hudson Bay provide a ΔR of +35 ± 18, higher than the database estimate, a negative ΔR of −59 ± 78 based on 35 shell and walrus samples, but within the 95.4% range. Western Greenland has a ΔR of −30 ± 97 in the 14CHRONO Marine20 Reservoir database, based on 56 mostly shell samples (there is one seal and one walrus); this agrees closely with the ΔR of −17 ± 47 which we calculated for the two walrus samples from that region. For the Foxe Basin, 44 shell samples and three walrus samples from the 14CHRONO Marine20 Reservoir database yield a ΔR of +164 ± 122, which is higher than that derived from the five walrus samples considered here (ΔR = +49 ± 29). It should be noted, however, that the range of ΔR from the Foxe Basin walrus samples is large (−10–+109), overlapping with the database estimate. Our six walrus samples from Eastern Greenland have a ΔR value of −26 ± 27, with a range from −82 to +29. In the reservoir database, 38 shell sample results are available for Eastern Greenland. These produce a ΔR of 84 ± 244 but with a very large ΔR range, which overlaps with our results.

In the reservoir database, 14 shell samples from Iceland have ΔR = −94 ± 76, which agrees well with our one walrus specimen from that island, which has ΔR = −49 ± 66. However, as this is only one sample, its range of possible ΔR values is broad (from −182 to +84). It should also be noted that the Icelandic walrus population is thought to have been extirpated in the Viking Age (Keighley et al., 2019); the individual we dated (killed in 1900) was probably a vagrant from another population, having unknown residency time in Icelandic waters.

Walruses generally migrate seasonally within the same region and return to the same localities each year (e.g. Born et al., 2005; Freitas et al., 2009; Mikkelsen et al., 2024; Solovyova et al., 2024; Wiig et al., 1996). Walruses occasionally cross vast distances, travelling to other regions, but this is thought to involve only a few individuals yearly (e.g. Chiacchio and Aae, 2024; Freitas et al., 2009; Pihlström et al., 2024; Wiig et al., 1996).

From Franz Josef Land, two shell measurements are available in the reservoir database (note that sample GX-19024, from the bivalve *Bathyarca glacialis*, is mis-attributed to whale in the database, see Pieńkowski et al., 2022). Both have negative ΔR values, −283 and −265, producing an average ΔR of −276 ± 31 (see also Pieńkowski et al., 2022). This is lower than the ΔR calculated for our two walrus samples from Franz Josef Land, −140 ± 47, but within the 95.4% range. It may be pertinent that the mollusc-derived ΔR for Svalbard (−61 ± 37) is also higher (less negative) than that from Franz Josef Land (Pieńkowski et al., 2022). Some migration of walruses is documented between the two archipelagoes (e.g. Mikkelsen et al., 2024), so walrus ΔR values in the Barents Sea may vary depending on the degree of feeding in each group of islands.

The exceptionally high ΔR value of our two samples from the Pacific (295 ± 47) is supported by 12 shell samples from the reservoir database, with ΔR = 305 ± 102. Our Pacific value is also comparable with the published ΔR of 289 ± 124 based on terrestrial versus marine (seal and walrus) sample pairs from archaeological contexts in coastal Chukotka and Alaska (Dury et al., 2022).

Overall, the results based on known-age samples indicate the importance of using population-specific ΔR values for walruses, especially in extreme cases such as the Pacific and Franz Josef Land, but to a lesser degree also in other areas. Where both shell- and walrus-derived ΔR values exist, species and population-specific walrus ΔR values are preferable. However, most walrus and shell data are broadly consistent, justifying use of shells where known-age walrus specimens do not exist. This would apply, for example, when calibrating radiocarbon dates for the Maritimes walrus of eastern Canada, which was extirpated in the 18th century CE, leaving no extant known-age specimens (McCaffrey, 2020; McLeod et al., 2014). Caution does remain necessary in locations where there are few or no known-date walrus samples and shell data indicate high intra-regional variability (e.g. East Greenland). The seasonal mobility of walruses should dampen some local variability in shell-based ΔR values, but high feeding-site fidelity (e.g. Mikkelsen et al., 2024) means that ΔR variability within walrus populations could nevertheless occur where baseline food-source values are heterogeneous.

### Calibration of radiocarbon dates on archaeological walrus finds from the Trondheim Region

The walrus rostra and ivory finds from archaeological contexts in Trondheim and its hinterland are presently interpreted as evidence of trade from the medieval Norse colonies of Greenland (Barrett et al., 2020; Ruiz-Puerta et al., 2024). This interpretation is based on aDNA and/or stable isotope provenancing of a subset of the relevant finds (including six of the 12 specimens that are radiocarbon dated in this paper: N10102, N37603, N32091, N29494, N203145 and N167587), showing that they probably derived from western/northwestern Greenland or the eastern Canadian Arctic. Yet two of the provenanced specimens (N37603 and N167587) are from archaeological contexts that postdate the traditionally accepted 15th-century CE abandonment of Norse Greenland and pre-date the subsequent Scandinavian return to the island in the 18th century (Christophersen and Nordeide, 1994; Gulløv, 2016; Nordeide, 2000). The question thus arises whether these are earlier objects that have been redeposited in later archaeological layers (i.e. they are residual), or if Norwegian trade with Greenland continued later than assumed.

To address this question, we used our pooled ΔR values for Greenland (excluding East Greenland) and the Canadian Arctic (17 ± 12) to calibrate the radiocarbon dates of these finds, plus 10 other walrus artefacts from Trondheim (three unstratified and seven from 12th and 13th-century contexts; Table 2, Figure 5). In the first instance, we calibrated the 12 archaeological dates as a single group without stratigraphic information (Table 4). In a second OxCal calibration, we added the original archaeological context dating (except for the two potentially residual specimens, which were treated as unstratified) in a simple Bayesian model. In cases where the archaeological date of the layer in which the sample was found is known, the walrus must have been killed before the deposition in this layer, and we used simple sequence models as for the historical specimens. The archaeological specimens derive from several excavations. Thus, their sequence order in the OxCal model is inferred from the chronology of their contexts (as determined by artefact dating, radiocarbon, dendrochronology and/or intra-site stratigraphy) rather than a single stratigraphic sequence (see Supplemental Material Table S2). Where no archaeological dating is available, the relevant cell in Table 2 is left blank and no sequence model was used for that sample. In result, there is variable agreement between the walrus radiocarbon and archaeological dates; the agreement indices (A) are below the recommended threshold value of 60 for five specimens: TRa-25883, TRa-25886, TRa-25178, TRa-25884 and TRa-25179, suggesting that some may be intrusive or residual rather than being in their primary depositional context. Less likely, some could alternatively derive from walruses having a different ΔR (see further below). Regardless, the calibrated results from both approaches are consistent with all 12 objects having derived from between the 11th and early 15th centuries (Table 4). This dating corresponds with the known medieval trade in walrus ivory between Greenland and Norway (Barrett, 2021; Barrett and Grav, 2025) and is before the mid-15th-century abandonment of Norse Greenland (Arneborg, 2024).

In a third calibration we excluded the predetermined ΔR of 17 ± 12, and instead employed code allowing OxCal to calculate ΔR using only the archaeological context dates for reference. The result is −110 ± 35 (Figure 6), lower than the ΔR calculated for the historical samples. At face value, the negative ΔR derived from the archaeological dating could indicate a smaller marine reservoir offset than we have calculated using historical specimens. However, the difference from a ΔR of 17 ± 12 is more likely to reflect the elapsed time between when the walruses were hunted and when the resulting raw materials reached their final archaeological context (after transport, storage, use, primary deposition and potentially secondary or tertiary redeposition). If so, the ΔR based on the known-age natural history specimens will be more reliable. Alternatively, a ΔR difference between 19th- to 20th-century and medieval walruses from the same region could indicate changes in feeding behaviour. This possibility can be explored by stable isotope (δ^13^C, δ^15^N, δ^34^S) analysis in future research (Dierickx et al., 2026b).

If the Trondheim finds are calibrated assuming a ΔR of −110 ± 35 (Table 4), the resulting date ranges are slightly older than when 17 ± 12 was employed (given the smaller marine reservoir adjustment of the raw radiocarbon determinations). However, regardless of which of the three calibration approaches is adopted, it is clear that all of the specimens dated relate to the centuries during which walrus ivory trade between the Norse colonies of Greenland and Norway is plausible based on existing archaeological and historical evidence (Arneborg, 2021).

## Conclusion

The large differences in ΔR values for the different populations show that it is important to consider the population of origin when radiocarbon dating walrus samples. This can be a challenge for samples of unknown provenance (e.g. archaeological samples). Provenance studies (e.g. using aDNA and stable isotopes) may thus be a prerequisite for accurate radiocarbon dating of walrus samples. In many instances, walrus and shell samples from the same localities produce similar ΔR values, indicating that shell data can often serve as an acceptable alternative where known-age walrus specimens do not exist, as is the case for extirpated populations. Caution is required, however, in regions with heterogeneous ΔR values based on shell or walrus samples. Using our ΔR results from known-date natural history specimens to calibrate radiocarbon dates on walrus artefacts from Trondheim it is possible to confirm that seemingly anomalous archaeological dates are due to redeposition of residual artefacts.

## Supplemental Material

sj-docx-1-hol-10.1177_09596836261468295 – Supplemental material for Walrus population-specific marine reservoir offsets (ΔR) for calibration of radiocarbon dates: Implications for Arctic chronologies and medieval trade Supplemental material, sj-docx-1-hol-10.1177_09596836261468295 for Walrus population-specific marine reservoir offsets (ΔR) for calibration of radiocarbon dates: Implications for Arctic chronologies and medieval trade by Bente Philippsen, Katrien Dierickx, Mohsen Falahati-Anbaran, Erin H. Kunisch, Thomas C. A. Royle, Richard Sabin, Eirik Sollid and James H. Barrett in The Holocene

sj-docx-2-hol-10.1177_09596836261468295 – Supplemental material for Walrus population-specific marine reservoir offsets (ΔR) for calibration of radiocarbon dates: Implications for Arctic chronologies and medieval trade Supplemental material, sj-docx-2-hol-10.1177_09596836261468295 for Walrus population-specific marine reservoir offsets (ΔR) for calibration of radiocarbon dates: Implications for Arctic chronologies and medieval trade by Bente Philippsen, Katrien Dierickx, Mohsen Falahati-Anbaran, Erin H. Kunisch, Thomas C. A. Royle, Richard Sabin, Eirik Sollid and James H. Barrett in The Holocene

sj-xlsx-1-hol-10.1177_09596836261468295 – Supplemental material for Walrus population-specific marine reservoir offsets (ΔR) for calibration of radiocarbon dates: Implications for Arctic chronologies and medieval trade Supplemental material, sj-xlsx-1-hol-10.1177_09596836261468295 for Walrus population-specific marine reservoir offsets (ΔR) for calibration of radiocarbon dates: Implications for Arctic chronologies and medieval trade by Bente Philippsen, Katrien Dierickx, Mohsen Falahati-Anbaran, Erin H. Kunisch, Thomas C. A. Royle, Richard Sabin, Eirik Sollid and James H. Barrett in The Holocene

## Data Availability

All raw data can be found in Supplemental Material File 1, containing Tables S1, S2 and S3. OxCal code is provided in Supplemental Material File 2.
