# Supplementary material for: Walrus population-specific marine reservoir offsets (ΔR) for calibration of radiocarbon dates: Implications for Arctic chronologies and medieval trade
Source: Holocene. 2026 Aug 15;36(11):1350–64. doi: 10.1177/09596836261468295 (PMC13716312; doi:10.1177/09596836261468295)
Supplement: Supplementary file 1 [file sj-docx-1-hol-10.1177_09596836261468295.docx]

## Supplemental Material 2. OxCal code for 𝛥R determinations by population or location and for calibration of the archaeological specimens from Trondheim and its hinterland

### 2.1.1 Baffin Bay, including date of death only

Plot()

{

Curve("Marine20", "marine20.14c");

Delta_R("BAF",U(-500,500));

Sequence("Year of death 1853")

{

Boundary("Start 1853");

R_Date("TRa-22945",649,12);

C_Date("1853",1853,2);

Boundary("End 1853");

};

Sequence("Year of death 1854")

{

Boundary("Start 1854");

Phase("1854 walrus")

{

R_Date("TRa-22943",725,15);

R_Date("TRa-22944",737,13);

};

C_Date("1854",1854,2);

Boundary("End 1854");

};

Sequence("Year of death 1895")

{

Boundary("Start 1895");

R_Date("TRa-25066",582,13);

C_Date("1895",1895,1);

Boundary("End 1895");

};

Sequence("Year of death 1896")

{

Boundary("Start 1896");

Phase("1896 walrus")

{

R_Date("TRa-25064",535,13);

R_Date("TRa-25069",582,13);

R_Date("TRa-25065",599,15);

R_Date("TRa-25068",603,14);

R_Date("TRa-25063",569,21);

};

C_Date("1896",1896,1);

Boundary("End 1896");

};

Sequence("Year of death 1926")

{

Boundary("Start 1926");

Phase("1926 walrus")

{

R_Date("TRa-25060",553,16);

R_Date("TRa-25061",563,13);

R_Date("TRa-25062",565,18);

};

C_Date("1926",1926,1);

Boundary("End 1926");

};

};

### 2.1.2 Baffin Bay, including date of death and estimated date of birth

Plot()

{

Curve("Marine20", "marine20.14c");

Delta_R("BAF",U(-500,500));

Sequence("Year of death 1853")

{

Boundary("Start 1853");

C_Date("1853 adult",1828,9);

R_Date("TRa-22945",649,12);

C_Date("1853",1853,2);

Boundary("End 1853");

};

Sequence("Year of death 1854")

{

Boundary("Start 1854");

C_Date("1854 adults",1829,9);

Phase("1854 walrus")

{

R_Date("TRa-22943",725,15);

R_Date("TRa-22944",737,13);

};

C_Date("1854",1854,2);

Boundary("End 1854");

};

Sequence("Year of death 1895")

{

Boundary("Start 1895");

C_Date("1895 adult",1870,8);

R_Date("TRa-25066",582,13);

C_Date("1895",1895,1);

Boundary("End 1895");

};

Sequence("Year of death 1896")

{

Boundary("Start 1896");

C_Date("1896 adults",1871,8);

Phase("1896 walrus")

{

R_Date("TRa-25064",535,13);

R_Date("TRa-25069",582,13);

R_Date("TRa-25065",599,15);

R_Date("TRa-25068",603,14);

R_Date("TRa-25063",569,21);

};

C_Date("1896",1896,1);

Boundary("End 1896");

};

Sequence("Year of death 1926 adults")

{

Boundary("Start 1926 adults");

C_Date("1926 adults DOB",1901,8);

Phase("1926 walrus")

{

R_Date("TRa-25060",553,16);

R_Date("TRa-25062",565,18);

};

C_Date("1926 adults DOD",1926,1);

Boundary("End 1926 adults");

};

Sequence("Year of death 1926 juvenile")

{

Boundary("Start 1926 juvenile");

C_Date("1926 juvenile DOB",1919,2);

R_Date("TRa-25061",563,13);

C_Date("1926 juvenile DOD",1926,1);

Boundary("End 1926 juvenile");

};

};

### 2.1.3 Baffin Bay, including date of death and estimated date of birth; pre-1950 dates from this study and Dyke et al. 2019

Plot()

{

Curve("Marine20", "marine20.14c");

Delta_R("BAF",U(-500,500));

Sequence("Year of death 1853")

{

Boundary("Start 1853");

C_Date("1853 adult",1828,9);

R_Date("TRa-22945",649,12);

C_Date("1853",1853,2);

Boundary("End 1853");

};

Sequence("Year of death 1854")

{

Boundary("Start 1854");

C_Date("1854 adults",1829,9);

Phase("1854 walrus")

{

R_Date("TRa-22943",725,15);

R_Date("TRa-22944",737,13);

};

C_Date("1854",1854,2);

Boundary("End 1854");

};

Sequence("Year of death 1895")

{

Boundary("Start 1895");

C_Date("1895 adult",1870,8);

R_Date("TRa-25066",582,13);

C_Date("1895",1895,1);

Boundary("End 1895");

};

Sequence("Year of death 1896")

{

Boundary("Start 1896");

C_Date("1896 adults",1871,8);

Phase("1896 walrus")

{

R_Date("TRa-25064",535,13);

R_Date("TRa-25069",582,13);

R_Date("TRa-25065",599,15);

R_Date("TRa-25068",603,14);

R_Date("TRa-25063",569,21);

};

C_Date("1896",1896,1);

Boundary("End 1896");

};

Sequence("Year of death 1924 ontology uncertain")

{

Boundary("Start 1924 ontology uncertain");

C_Date("1924 ontology uncertain DOB",1904,10);

R_Date("UCIAMS-168854",755,15);

C_Date("1924 ontology uncertain DOD",1924,1);

Boundary("End 1924 ontology uncertain");

};

Sequence("Year of death 1926 adults")

{

Boundary("Start 1926 adults");

C_Date("1926 adults DOB",1901,8);

Phase("1926 walrus")

{

R_Date("TRa-25060",553,16);

R_Date("TRa-25062",565,18);

};

C_Date("1926 adults DOD",1926,1);

Boundary("End 1926 adults");

};

Sequence("Year of death 1926 juvenile")

{

Boundary("Start 1926 juvenile");

C_Date("1926 juvenile DOB",1919,2);

R_Date("TRa-25061",563,13);

C_Date("1926 juvenile DOD",1926,1);

Boundary("End 1926 juvenile");

};

};

### 2.2.1 West Greenland, including date of death only

Plot()

{

Curve("Marine20", "marine20.14c");

Delta_R("WGL",U(-500,500));

Sequence("Year of death 1898")

{

Boundary("Start 1898");

R_Date("TRa-25591",604,13);

C_Date("1898",1898,1);

Boundary("End 1898");

};

Sequence("Year of death 1895")

{

Boundary("Start 1895");

R_Date("TRa-25067",611,20);

C_Date("1895",1895,1);

Boundary("End 1895");

};

};

### 2.2.2 West Greenland, including date of death and estimated date of birth

Plot()

{

Curve("Marine20", "marine20.14c");

Delta_R("WGL",U(-500,500));

Sequence("Year of death 1898")

{

Boundary("Start 1898");

C_Date("1898 adult DOB",1873,8);

R_Date("TRa-25591",604,13);

C_Date("1898 DOD",1898,1);

Boundary("End 1898");

};

Sequence("Year of death 1895")

{

Boundary("Start 1895");

C_Date("1895 juvenile DOB",1888,2);

R_Date("TRa-25067",611,20);

C_Date("1895 DOD",1895,1);

Boundary("End 1895");

};

};

### 2.3.1 Foxe Basin, including date of death only

Plot()

{

Curve("Marine20", "marine20.14c");

Delta_R("SFO",U(-500,500));

Sequence("Year of death 1922")

{

Boundary("Start 1922");

Phase("phase 1922")

{

R_Date("TRa-25596",687,16);

R_Date("TRa-25595",646,18);

};

C_Date("1922",1922,1);

Boundary("End 1922");

};

};

### 2.3.2 Foxe Basin, including date of death and estimated date of birth

Plot()

{

Curve("Marine20", "marine20.14c");

Delta_R("SFO",U(-500,500));

Sequence("Year of death 1922")

{

Boundary("Start 1922");

C_Date("1922 juveniles DOB",1915,2);

Phase("phase 1922")

{

R_Date("TRa-25596",687,16);

R_Date("TRa-25595",646,18);

};

C_Date("1922 juveniles DOD",1922,1);

Boundary("End 1922");

};

};

### 2.3.3 Foxe Basin, including date of death and estimated date of birth; pre-1950 dates from this study and Dyke et al. 2019

Plot()

{

Curve("Marine20", "marine20.14c");

Delta_R("SFO",U(-500,500));

Sequence("Year of death 1922")

{

Boundary("Start 1922");

C_Date("1922 juveniles DOB",1915,2);

Phase("phase 1922")

{

R_Date("TRa-25596",687,16);

R_Date("TRa-25595",646,18);

};

C_Date("1922 juveniles DOD",1922,1);

Boundary("End 1922");

};

Sequence("Year of death 1949")

{

Boundary("Start 1949");

C_Date("1949 ontology unknown DOB",1929,10);

Phase("phase 1949")

{

R_Date("UCIAMS-168835",595,15);

R_Date("UCIAMS-168834",655,15);

R_Date("UCIAMS-168833",685,15);

};

C_Date("1949 ontology unknown DOD",1949,1);

Boundary("End 1949");

};

};

### 2.4.1 Hudson Strait/Hudson Bay, including date of death only

Plot()

{

Curve("Marine20", "marine20.14c");

Delta_R("HUD",U(-500,500));

Sequence("Year of death 1885")

{

Boundary("Start 1885");

Phase("1885 walrus")

{

R_Date("TRa-24149",671,20);

R_Date("TRa-24150",639,13);

};

C_Date("1885",1885,2);

Boundary("End 1885");

};

Sequence("Year of death 1886")

{

Boundary("Start 1886");

R_Date("TRa-24148",609,12);

C_Date("1886",1886,1);

Boundary("End 1886");

};

Sequence("Year of death 1910")

{

Boundary("Start 1910");

R_Date("TRa-24151",615,12);

C_Date("1910",1910,1);

Boundary("End 1910");

};

};

### 2.4.2 Hudson Strait/Hudson Bay, including date of death and estimated date of birth

Plot()

{

Curve("Marine20", "marine20.14c");

Delta_R("HUD",U(-500,500));

Sequence("Year of death 1885")

{

Boundary("Start 1885");

C_Date("1885 juveniles DOB",1878,3);

Phase("1885 walrus")

{

R_Date("TRa-24149",671,20);

R_Date("TRa-24150",639,13);

};

C_Date("1885 DOD",1885,2);

Boundary("End 1885");

};

Sequence("Year of death 1886")

{

Boundary("Start 1886");

C_Date("1886 adult DOB",1861,8);

R_Date("TRa-24148",609,12);

C_Date("1886 DOD",1886,1);

Boundary("End 1886");

};

Sequence("Year of death 1910")

{

Boundary("Start 1910");

C_Date("1910 adult DOB",1885,8);

R_Date("TRa-24151",615,12);

C_Date("1910 DOD",1910,1);

Boundary("End 1910");

};

};

### 2.4.3 Hudson Strait/Hudson Bay, including date of death and estimated date of birth; pre-1950 dates from this study and Dyke et al. 2019

Plot()

{

Curve("Marine20", "marine20.14c");

Delta_R("HUD",U(-500,500));

Sequence("Year of death 1885 ontology uncertain")

{

Boundary("Start 1885 ontology uncertain");

C_Date("1885 ontology uncertain DOB",1865,10);

R_Date("UCIAMS-168836",670,15);

C_Date("1885 ontology uncertain DOD",1885,1);

Boundary("End 1885 ontology uncertain");

};

Sequence("Year of death 1885 juveniles")

{

Boundary("Start 1885 juveniles");

C_Date("1885 juveniles DOB",1878,3);

Phase("1885 juvenile walruses")

{

R_Date("TRa-24149",671,20);

R_Combine("CMH36")

{

R_Date("CMH36_TRa-24150",639,13);

R_Date("CMH36_UCIAMS-168850",665,15);

};

};

C_Date("1885 juveniles DOD",1885,2);

Boundary("End 1885 juveniles");

};

Sequence("Year of death 1886 adult")

{

Boundary("Start 1886 adult");

C_Date("1886 adult DOB",1861,8);

R_Date("TRa-24148",609,12);

C_Date("1886 adult DOD",1886,1);

Boundary("End 1886 adult");

};

Sequence("Year of death 1886 ontology uncertain")

{

Boundary("Start 1886 ontology uncertain");

C_Date("1886 ontology uncertain DOB",1866,10);

R_Date("UCIAMS-168857",630,15);

C_Date("1886 ontology uncertain DOD",1886,1);

Boundary("End 1886 ontology uncertain");

};

Sequence("Year of death 1910")

{

Boundary("Start 1910");

C_Date("1910 adult DOB",1885,8);

R_Date("TRa-24151",615,12);

C_Date("1910 DOD",1910,1);

Boundary("End 1910");

};

Sequence("Year of death 1923")

{

Boundary("Start 1923 ontology uncertain");

C_Date("1923 ontology uncertain DOB",1903,10);

R_Date("UCIAMS-168851",595,15);

C_Date("1923 ontology uncertain DOD",1923,1);

Boundary("End 1923 ontology uncertain");

};

Sequence("Year of death 1924")

{

Boundary("Start 1924 ontology uncertain");

C_Date("1924 ontology uncertain DOB",1904,10);

Phase("phase 1924 ontology uncertain")

{

R_Date("UCIAMS-168852",660,15);

R_Date("UCIAMS-168855",670,15);

R_Date("UCIAMS-168853",690,15);

R_Date("UCIAMS-168856",695,15);

};

C_Date("1924 ontology uncertain DOD",1924,1);

Boundary("End 1924 ontology uncertain");

};

Sequence("Year of death 1928")

{

Boundary("Start 1928 ontology uncertain");

C_Date("1928 ontology uncertain DOB",1908,10);

Phase("phase 1928 ontology uncertain")

{

R_Date("UCIAMS-185717",625,15);

R_Date("UCIAMS-168831",705,15);

};

C_Date("1928 ontology uncertain DOD",1928,1);

Boundary("End 1928 ontology uncertain");

};

Sequence("Year of death 1945")

{

Boundary("Start 1945 ontology uncertain");

C_Date("1945 ontology uncertain DOB",1925,10);

R_Date("UCIAMS-168832",635,15);

C_Date("1945 ontology uncertain DOD",1945,1);

Boundary("End 1945 ontology uncertain");

};

};

### 2.5.1 Pooled Greenland (excluding East Greenland) and Canadian Arctic, including date of death only

Plot()

{

Curve("Marine20", "marine20.14c");

Delta_R("Greenl_Can_Arct",U(-500,500));

Sequence("Baffin Year of death 1853")

{

Boundary("Start BAF 1853");

R_Date("TRa-22945",649,12);

C_Date("1853",1853,2);

Boundary("End BAF 1853");

};

Sequence("Baffin Year of death 1854")

{

Boundary("Start BAF 1854");

Phase("BAF 1854 walrus")

{

R_Date("TRa-22943",725,15);

R_Date("TRa-22944",737,13);

};

C_Date("1854",1854,2);

Boundary("End BAF 1854");

};

Sequence("Hudson Strait Year of death 1885")

{

Boundary("Start HUD 1885");

Phase("HUD 1885 walrus")

{

R_Date("TRa-24149",671,20);

R_Date("TRa-24150",639,13);

};

C_Date("1885",1885,2);

Boundary("End HUD 1885");

};

Sequence("Hudson Strait Year of death 1886")

{

Boundary("Start HUD 1886");

R_Date("TRa-24148",609,12);

C_Date("1886",1886,1);

Boundary("End HUD 1886");

};

Sequence("Baffin Year of death 1895")

{

Boundary("Start BAF 1895");

R_Date("TRa-25066",582,13);

C_Date("Baffin 1895",1895,1);

Boundary("End BAF 1895");

};

Sequence("Western Greenland Year of death 1895")

{

Boundary("Start WG 1895");

R_Date("TRa-25067",611,20);

C_Date("1895",1895,1);

Boundary("End WG 1895");

};

Sequence("Baffin Year of death 1896")

{

Boundary("Start BAF 1896");

Phase("BAF 1896 walrus")

{

R_Date("TRa-25064",535,13);

R_Date("TRa-25069",582,13);

R_Date("TRa-25065",599,15);

R_Date("TRa-25068",603,14);

R_Date("TRa-25063",569,21);

};

C_Date("1896",1896,1);

Boundary("End BAF 1896");

};

Sequence("Western Greenland Year of death 1898")

{

Boundary("Start WG 1898");

R_Date("TRa-25591",604,13);

C_Date("1898",1898,1);

Boundary("End WG 1898");

};

Sequence("Hudson Strait_Hudson Bay Year of death 1910")

{

Boundary("Start HUD 1910");

R_Date("TRa-24151",615,12);

C_Date("1910",1910,1);

Boundary("End HUD 1910");

};

Sequence("Foxe Basin Year of death 1922")

{

Boundary("Start FOX 1922");

Phase("FOX 1922 walrus")

{

R_Date("TRa-25596",687,16);

R_Date("TRa-25595",646,18);

};

C_Date("Foxe 1922",1922,1);

Boundary("End FOX 1922");

};

Sequence("Baffin Year of death 1926")

{

Boundary("Start BAF 1926");

Phase("BAF 1926 walrus")

{

R_Date("TRa-25060",553,16);

R_Date("TRa-25061",563,13);

R_Date("TRa-25062",565,18);

};

C_Date("1926",1926,1);

Boundary("End BAF 1926");

};

};

### 2.5.2 Pooled Greenland (excluding East Greenland) and Canadian Arctic, including date of death and estimated date of birth

Plot()

{

Curve("Marine20", "marine20.14c");

Delta_R("BAF",U(-500,500));

Sequence("BAF Year of death 1853")

{

Boundary("Start BAF 1853");

C_Date("BAF 1853 adult DOB",1828,9);

R_Date("TRa-22945",649,12);

C_Date("BAF 1853 adult DOD",1853,2);

Boundary("End BAF 1853");

};

Sequence("BAF Year of death 1854")

{

Boundary("Start BAF 1854");

C_Date("BAF 1854 adults DOB",1829,9);

Phase("phase BAF 1854 walrus")

{

R_Date("TRa-22943",725,15);

R_Date("TRa-22944",737,13);

};

C_Date("BAF 1854 adults DOD",1854,2);

Boundary("End BAF 1854");

};

Sequence("HUD Year of death 1885")

{

Boundary("Start HUD 1885");

C_Date("HUD 1885 juveniles DOB",1878,3);

Phase("phase HUD 1885 walrus")

{

R_Date("TRa-24149",671,20);

R_Date("TRa-24150",639,13);

};

C_Date("HUD 1885 juveniles DOD",1885,2);

Boundary("End HUD 1885");

};

Sequence("HUD Year of death 1886")

{

Boundary("Start HUD 1886");

C_Date("HUD 1886 adult DOB",1861,8);

R_Date("TRa-24148",609,12);

C_Date("HUD 1886 adult DOD",1886,1);

Boundary("End HUD 1886");

};

Sequence("BAF Year of death 1895 adult")

{

Boundary("Start BAF 1895");

C_Date("BAF 1895 adult DOB",1870,8);

R_Date("TRa-25066",582,13);

C_Date("BAF 1895 adult DOD",1895,1);

Boundary("End BAF 1895");

};

Sequence("WGL Year of death 1895 juvenile")

{

Boundary("Start WGL 1895");

C_Date("WGL 1895 juvenile DOB",1888,2);

R_Date("TRa-25067",611,20);

C_Date("WGL 1895 juvenile DOD",1895,1);

Boundary("End WGL 1895");

};

Sequence("BAF Year of death 1896")

{

Boundary("Start BAF 1896");

C_Date("BAF 1896 adults DOB",1871,8);

Phase("phase BAF 1896 walrus")

{

R_Date("TRa-25064",535,13);

R_Date("TRa-25069",582,13);

R_Date("TRa-25065",599,15);

R_Date("TRa-25068",603,14);

R_Date("TRa-25063",569,21);

};

C_Date("BAF 1896 adults DOD",1896,1);

Boundary("End BAF 1896");

};

Sequence("WGL Year of death 1898")

{

Boundary("Start WGL 1898");

C_Date("WGL 1898 adult DOB",1873,8);

R_Date("TRa-25591",604,13);

C_Date("WGL 1898 DOD",1898,1);

Boundary("End WGL 1898");

};

Sequence("HUD Year of death 1910")

{

Boundary("Start HUD 1910");

C_Date("HUD 1910 adult DOB",1885,8);

R_Date("TRa-24151",615,12);

C_Date("HUD 1910 adult DOD",1910,1);

Boundary("End HUD 1910");

};

Sequence("FOX Year of death 1922")

{

Boundary("Start FOX 1922");

C_Date("FOX 1922 juveniles DOB",1915,2);

Phase("phase FOX 1922")

{

R_Date("TRa-25596",687,16);

R_Date("TRa-25595",646,18);

};

C_Date("FOX 1922 juveniles DOD",1922,1);

Boundary("End FOX 1922");

};

Sequence("BAF Year of death 1926 adults")

{

Boundary("Start BAF 1926 adults");

C_Date("BAF 1926 adults DOB",1901,8);

Phase("phase BAF 1926 walrus")

{

R_Date("TRa-25060",553,16);

R_Date("TRa-25062",565,18);

};

C_Date("BAF 1926 adults DOD",1926,1);

Boundary("End BAF 1926 adults");

};

Sequence("BAF Year of death 1926 juvenile")

{

Boundary("Start BAF 1926 juvenile");

C_Date("BAF 1926 juvenile DOB",1919,2);

R_Date("TRa-25061",563,13);

C_Date("BAF 1926 juvenile DOD",1926,1);

Boundary("End BAF 1926 juvenile");

};

};

### 2.5.3 Pooled Greenland (excluding East Greenland) and Canadian Arctic, including date of death and estimated date of birth; pre-1950 dates from this study and Dyke et al. 2019

Plot()

{

Curve("Marine20", "marine20.14c");

Delta_R("Greenl_Can_Arct",U(-500,500));

Sequence("BAF Year of death 1853")

{

Boundary("Start BAF 1853");

C_Date("BAF 1853 adult DOB",1828,9);

R_Date("TRa-22945",649,12);

C_Date("BAF 1853 adult DOD",1853,2);

Boundary("End BAF 1853");

};

Sequence("BAF Year of death 1854")

{

Boundary("Start BAF 1854");

C_Date("BAF 1854 adults DOB",1829,9);

Phase("phase BAF 1854 walrus")

{

R_Date("TRa-22943",725,15);

R_Date("TRa-22944",737,13);

};

C_Date("BAF 1854 adults DOD",1854,2);

Boundary("End BAF 1854");

};

Sequence("HUD Year of death 1885 ontology uncertain")

{

Boundary("Start HUD 1885 ontology uncertain");

C_Date("HUD 1885 ontology uncertain DOB",1865,10);

R_Date("UCIAMS-168836",670,15);

C_Date("HUD 1885 ontology uncertain DOD",1885,1);

Boundary("End HUD 1885 ontology uncertain");

};

Sequence("HUD Year of death 1885 juveniles")

{

Boundary("Start HUD 1885 juveniles");

C_Date("HUD 1885 juveniles DOB",1878,3);

Phase("phase HUD 1885 juvenile walruses")

{

R_Date("TRa-24149",671,20);

R_Combine("CMH36")

{

R_Date("CMH36_TRa-24150",639,13);

R_Date("CMH36_UCIAMS-168850",665,15);

};

};

C_Date("HUD 1885 juveniles DOD",1885,2);

Boundary("End HUD 1885 juveniles");

};

Sequence("HUD Year of death 1886 adult")

{

Boundary("Start HUD 1886 adult");

C_Date("HUD 1886 adult DOB",1861,8);

R_Date("TRa-24148",609,12);

C_Date("HUD 1886 adult DOD",1886,1);

Boundary("End HUD 1886 adult");

};

Sequence("HUD Year of death 1886 ontology uncertain")

{

Boundary("Start HUD 1886 ontology uncertain");

C_Date("HUD 1886 ontology uncertain DOB",1866,10);

R_Date("UCIAMS-168857",630,15);

C_Date("HUD 1886 ontology uncertain DOD",1886,1);

Boundary("End HUD 1886 ontology uncertain");

};

Sequence("BAF Year of death 1895 adult")

{

Boundary("Start BAF 1895");

C_Date("BAF 1895 adult DOB",1870,8);

R_Date("TRa-25066",582,13);

C_Date("BAF 1895 adult DOD",1895,1);

Boundary("End BAF 1895");

};

Sequence("WGL Year of death 1895 juvenile")

{

Boundary("Start WGL 1895");

C_Date("WGL 1895 juvenile DOB",1888,2);

R_Date("TRa-25067",611,20);

C_Date("WGL 1895 juvenile DOD",1895,1);

Boundary("End WGL 1895");

};

Sequence("BAF Year of death 1896")

{

Boundary("Start BAF 1896");

C_Date("BAF 1896 adults DOB",1871,8);

Phase("phase BAF 1896 walrus")

{

R_Date("TRa-25064",535,13);

R_Date("TRa-25069",582,13);

R_Date("TRa-25065",599,15);

R_Date("TRa-25068",603,14);

R_Date("TRa-25063",569,21);

};

C_Date("BAF 1896 adults DOD",1896,1);

Boundary("End BAF 1896");

};

Sequence("WGL Year of death 1898")

{

Boundary("Start WGL 1898");

C_Date("WGL 1898 adult DOB",1873,8);

R_Date("TRa-25591",604,13);

C_Date("WGL 1898 DOD",1898,1);

Boundary("End WGL 1898");

};

Sequence("HUD Year of death 1910")

{

Boundary("Start HUD 1910");

C_Date("HUD 1910 adult DOB",1885,8);

R_Date("TRa-24151",615,12);

C_Date("HUD 1910 adult DOD",1910,1);

Boundary("End HUD 1910");

};

Sequence("FOX Year of death 1922")

{

Boundary("Start FOX 1922");

C_Date("FOX 1922 juveniles DOB",1915,2);

Phase("phase FOX 1922")

{

R_Date("TRa-25596",687,16);

R_Date("TRa-25595",646,18);

};

C_Date("FOX 1922 juveniles DOD",1922,1);

Boundary("End FOX 1922");

};

Sequence("HUD Year of death 1923")

{

Boundary("Start HUD 1923 ontology uncertain");

C_Date("HUD 1923 ontology uncertain DOB",1903,10);

R_Date("UCIAMS-168851",595,15);

C_Date("HUD 1923 ontology uncertain DOD",1923,1);

Boundary("End HUD 1923 ontology uncertain");

};

Sequence("BAF Year of death 1924 ontology uncertain")

{

Boundary("Start BAF 1924 ontology uncertain");

C_Date("BAF 1924 ontology uncertain DOB",1904,10);

R_Date("UCIAMS-168854",755,15);

C_Date("BAF 1924 ontology uncertain DOD",1924,1);

Boundary("End BAF 1924 ontology uncertain");

};

Sequence("HUD Year of death 1924 ontology uncertain")

{

Boundary("Start HUD 1924 ontology uncertain");

C_Date("HUD 1924 ontology uncertain DOB",1904,10);

Phase("phase HUD 1924 ontology uncertain")

{

R_Date("UCIAMS-168852",660,15);

R_Date("UCIAMS-168855",670,15);

R_Date("UCIAMS-168853",690,15);

R_Date("UCIAMS-168856",695,15);

};

C_Date("HUD 1924 ontology uncertain DOD",1924,1);

Boundary("End HUD 1924 ontology uncertain");

};

Sequence("BAF Year of death 1926 adults")

{

Boundary("Start BAF 1926 adults");

C_Date("BAF 1926 adults DOB",1901,8);

Phase("phase BAF 1926 walrus")

{

R_Date("TRa-25060",553,16);

R_Date("TRa-25062",565,18);

};

C_Date("BAF 1926 adults DOD",1926,1);

Boundary("End BAF 1926 adults");

};

Sequence("BAF Year of death 1926 juvenile")

{

Boundary("Start BAF 1926 juvenile");

C_Date("BAF 1926 juvenile DOB",1919,2);

R_Date("TRa-25061",563,13);

C_Date("BAF 1926 juvenile DOD",1926,1);

Boundary("End BAF 1926 juvenile");

};

Sequence("HUD Year of death 1928")

{

Boundary("StartHUD 1928 ontology uncertain");

C_Date("HUD 1928 ontology uncertain DOB",1908,10);

Phase("phase HUD 1928 ontology uncertain")

{

R_Date("UCIAMS-185717",625,15);

R_Date("UCIAMS-168831",705,15);

};

C_Date("HUD 1928 ontology uncertain DOD",1928,1);

Boundary("End HUD 1928 ontology uncertain");

};

Sequence("HUD Year of death 1945")

{

Boundary("Start HUD 1945 ontology uncertain");

C_Date("HUD 1945 ontology uncertain DOB",1925,10);

R_Date("UCIAMS-168832",635,15);

C_Date("HUD 1945 ontology uncertain DOD",1945,1);

Boundary("End HUD 1945 ontology uncertain");

};

Sequence("FOX Year of death 1949")

{

Boundary("Start FOX 1949");

C_Date("FOX 1949 ontology unknown DOB",1929,10);

Phase("phase FOX 1949")

{

R_Date("UCIAMS-168835",595,15);

R_Date("UCIAMS-168834",655,15);

R_Date("UCIAMS-168833",685,15);

};

C_Date("FOX 1949 ontology unknown DOD",1949,1);

Boundary("End FOX 1949");

};

};

### 2.6.1 East Greenland, including date of death only

Plot()

{

Curve("Marine20", "marine20.14c");

Delta_R("EGL",U(-500,500));

Sequence("Year of death 1924")

{

Boundary("Start 1924");

R_Date("TRa-25593",569,13);

C_Date("1924",1924,1);

Boundary("End 1924");

};

Sequence("Year of death 1927")

{

Boundary("Start 1927");

R_Date("TRa-25587",605,23);

C_Date("1927",1927,1);

Boundary("End 1927");

};

Sequence("Year of death 1929")

{

Boundary("Start 1929");

Phase("1929 walruses")

{

R_Date("TRa-25585",561,16);

R_Date("TRa-25588",558,16);

R_Date("TRa-25586",567,15);

};

C_Date("1929 DOD",1929,3);

Boundary("End 1929");

};

Sequence("Year of death 1909")

{

Boundary("Start 1909");

R_Date("TRa-25589",631,21);

C_Date("1909",1909,4);

Boundary("End 1909");

};

};

### 2.6.2 East Greenland, including date of death and estimated date of birth

Plot()

{

Curve("Marine20", "marine20.14c");

Delta_R("EGL",U(-500,500));

Sequence("Year of death 1924")

{

Boundary("Start 1924");

C_Date("1924 adult DOB",1899,8);

R_Date("TRa-25593",569,13);

C_Date("1924 DOD",1924,1);

Boundary("End 1924");

};

Sequence("Year of death 1927")

{

Boundary("Start 1927");

C_Date("1927 adult DOB",1902,8);

R_Date("TRa-25587",605,23);

C_Date("1927 DOD",1927,1);

Boundary("End 1927");

};

Sequence("Year of death 1929 adults")

{

Boundary("Start 1929 adults");

C_Date("1929 adults DOB",1904,10);

Phase("1929 adults")

{

R_Date("TRa-25585",561,16);

R_Date("TRa-25588",558,16);

};

C_Date("1929 adults DOD",1929,3);

Boundary("End 1929 adults");

};

Sequence("Year of death 1929 juvenile")

{

Boundary("Start 1929 juvenile");

C_Date("1929 juvenile DOB",1922,2);

R_Date("TRa-25586",567,15);

C_Date("1929 juvenile DOD",1929,1);

Boundary("End 1929 juvenile");

};

Sequence("Year of death 1909")

{

Boundary("Start 1909");

C_Date("1909 adult DOB",1884,11);

R_Date("TRa-25589",631,21);

C_Date("1909 DOD",1909,4);

Boundary("End 1909");

};

};

### 2.7.1 Iceland, including date of death only

Plot()

{

Curve("Marine20", "marine20.14c");

Delta_R("ICE",U(-500,500));

Sequence("Year of death 1900")

{

Boundary("Start 1900");

R_Date("TRa-25590",576,14);

C_Date("1900",1900,1);

Boundary("End 1900");

};

};

2.7.2 Iceland, including date of death and estimated date of birth

### Plot()

{

Curve("Marine20", "marine20.14c");

Delta_R("ICE",U(-500,500));

Sequence("Year of death 1900")

{

Boundary("Start 1900");

C_Date("1900 adult DOB",1875,8);

R_Date("TRa-25590",576,14);

C_Date("1900 DOD",1900,1);

Boundary("End 1900");

};

};

### 2.8.1 Franz Josef Land, including date of death only

Plot()

{

Curve("Marine20", "marine20.14c");

Delta_R("FJL",U(-500,500));

Sequence("Year of death 1894 to 1897")

{

Boundary("Start 1897");

Phase("phase 1897")

{

R_Date("TRa-22952",472,15);

R_Date("TRa-22947",494,15);

};

C_Date("1897",1897,4);

Boundary("End 1897");

};

};

### 2.8.2 Franz Josef Land, including date of death and estimated date of birth

Plot()

{

Curve("Marine20", "marine20.14c");

Delta_R("FJL",U(-500,500));

Sequence("Year of death 1894 to 1897 adult")

{

Boundary("Start 1897 adult");

C_Date("1897 adult DOB",1872,11);

R_Date("TRa-22947",494,15);

C_Date("1897 adult DOD",1897,4);

Boundary("End 1897 adult");

};

Sequence("Year of death 1894 to 1897 neonatal")

{

Boundary("Start 1897 neonatal");

C_Date("1897 neonatal DOB",1895,4);

R_Date("TRa-22952",472,15);

C_Date("1897 neonatal DOD",1897,4);

Boundary("End 1897 neonatal");

};

};

### 2.9.1 Pacific, including date of death only

Plot()

{

Curve("Marine20", "marine20.14c");

Delta_R("PAC",U(-500,500));

Sequence("Year of death 1865")

{

Boundary("Start 1865");

R_Date("TRa-22951",843,13);

C_Date("1865",1865,1);

Boundary("End 1865");

};

Sequence("Year of death 1897")

{

Boundary("Start 1897");

R_Date("TRa-22950",1026,16);

C_Date("1897",1897,2);

Boundary("End 1897");

};

};

### 2.9.2 Pacific, including date of death and estimated date of birth

Plot()

{

Curve("Marine20", "marine20.14c");

Delta_R("PAC",U(-500,500));

Sequence("Year of death 1865")

{

Boundary("Start 1865");

C_Date("1865 juvenile DOB",1858,2);

R_Date("TRa-22951",843,13);

C_Date("1865 DOD",1865,1);

Boundary("End 1865");

};

Sequence("Year of death 1897")

{

Boundary("Start 1897");

C_Date("1897 adult DOB",1872,9);

R_Date("TRa-22950",1026,16);

C_Date("1897 DOD",1897,2);

Boundary("End 1897");

};

};

### 2.10.1 Archaeological samples; calibration using ΔR of 17±12 with no constraints based on archaeological dating

Plot()

{

Curve("Marine20", "marine20.14c");

Delta_R("Pooled", 17,12);

R_Date("TRa-25177",1229,13);

R_Date("TRa-25175",1389,21);

R_Date("TRa-25176",1388,14);

R_Date("TRa-25178",1363,20);

R_Date("TRa-25883",1226,13);

R_Date("TRa-25884",1321,12);

R_Date("TRa-25886",1364,34);

R_Date("TRa-25887",1255,11);

R_Date("TRa-25885",1351,12);

R_Date("TRa-25179",1312,13);

R_Date("TRa-25888",1313,14);

R_Date("TRa-25180",1399,16);

};

### 2.10.2 Calibration including archaeological dating

This code includes both options mentioned in the text, 1) using the ΔR of 17±12 calculated for historical specimens from Greenland (excluding Eastern Greenland) and the Canadian Arctic, together with archaeological dating of the archaeological find contexts and 2) allowing OxCal to estimate a ΔR based on the nominal dating of the archaeological find contexts (the two options can be swapped depending on which Delta_R command is commented out).

Plot()

{

Curve("Marine20", "marine20.14c");

//Delta_R("Modern DeltaR W Greenland and Canadian Arctic", 17,12);

Delta_R("DeltaR old walrus",U(-400,400));

Sequence("Trondheim 1111")

{

Boundary("Start Trondheim 1111");

R_Date("TRa-25180", 1399, 16);

C_Date("1111", 1111, 1);

Boundary("End Trondheim 1111");

};

Label("Trondheim post 1600:");

R_Date("TRa-25175", 1389, 21);

Sequence("Trondheim 1225-1275")

{

Boundary("Start Trondheim 1225-1275");

Phase("Phase Trondheim 1225-1275")

{

R_Date("TRa-25176", 1388, 14);

R_Date("TRa-25883", 1226, 13);

};

C_Date("1225-1275", 1250, 13);

Boundary("End Trondheim 1225-1275");

};

Sequence("Trondheim 1100-1150")

{

Boundary("Start Trondheim 1100-1150");

R_Date("TRa-25886", 1364, 34);

C_Date("1100-1150", 1125, 13);

Boundary("End Trondheim 1100-1150");

};

Sequence("Trondheim 1150-1175")

{

Boundary("Start Trondheim 1150-1175");

R_Date("TRa-25178", 1363, 20);

C_Date("1150-1175", 1163, 6);

Boundary("End Trondheim 1150-1175");

};

R_Date("TRa-25885", 1351, 12);

Sequence("Trondheim 1175-1225")

{

Boundary("Start Trondheim 1175-1225");

R_Date("TRa-25884", 1321, 12);

C_Date("1175-1225", 1200, 13);

Boundary("End Trondheim 1175-1225");

};

R_Date("TRa-25888", 1313, 14);

Sequence("Trondheim 12th century")

{

Boundary("Start Trondheim 12th century");

R_Date("TRa-25179", 1312, 13);

C_Date("1100-1200", 1150, 25);

Boundary("End Trondheim 12th century");

};

R_Date("TRa-25887", 1255, 11);

R_Date("TRa-25177", 1229, 13);

};
